# Epidemiologic Patterns of Ross River Virus Disease in Queensland, Australia, 2001–2011

**DOI:** 10.4269/ajtmh.13-0455

**Published:** 2014-07-02

**Authors:** Weiwei Yu, Kerrie Mengersen, Pat Dale, John S. Mackenzie, Ghasem (Sam) Toloo, Xiaoyu Wang, Shilu Tong

**Affiliations:** School of Public Health and Social Work, Institute of Health and Biomedical Innovation; Disciplines of Mathematical Sciences, Faculty of Science and Technology Queensland University of Technology, Brisbane, Australia; Environmental Futures Centre, Griffith School of Environment, Griffith University, Nathan, Queensland, Australia; Faculty of Health Sciences, Curtin University, Perth, Western Australia, Australia; School of Chemistry and Molecular Biosciences, University of Queensland, Brisbane, Queensland, Australia; Burnet Institute, Melbourne, Victoria, Australia

## Abstract

Ross River virus (RRV) infection is a debilitating disease that has a significant impact on population health, economic productivity, and tourism in Australia. This study examined epidemiologic patterns of RRV disease in Queensland, Australia, during January 2001–December 2011 at a statistical local area level. Spatio-temporal analyses were used to identify the patterns of the disease distribution over time stratified by age, sex, and space. The results show that the mean annual incidence was 54 per 100,000 persons, with a male:female ratio of 1:1.1. Two space-time clusters were identified: the areas adjacent to Townsville, on the eastern coast of Queensland, and the southeast areas. Thus, although public health intervention should be considered across all areas in which RRV occurs, it should specifically focus on high-risk regions, particularly during summer and autumn to reduce the social and economic impacts of RRV infection.

## Introduction

Ross River virus (RRV) disease is the most widely spread mosquito-borne disease in Australia. In 2011, RRV infection accounted for 63% (5,149) of all reported mosquito-borne disease notifications.^1^ The notifications from Queensland accounted for 23% of all RRV cases in Australia.^1^

Ross River virus is an alphavirus that was first identified at Townsville in northern Queensland in 1959.^2^ This virus causes a non-fatal, but prolonged and debilitating disease known as epidemic polyarthritis or RRV disease. The disease syndrome is characterized by headache, fever, rash, lethargy and muscle and joint pain. The epidemiology of RRV disease is complex because transmission cycles are driven by various mosquito species and vertebrate hosts within a variety of disparate geoclimatic regions.^3^–^5^ More than 40 mosquito species have been implicated as vectors of RRV.^5^,^6^ Currently, there is no vaccination and specific treatment for RRV disease.^7^

The costs of healthcare for RRV disease are high and, taken together with productivity loss, were estimated to be between AU$1,018 and AU$1,180 per person.^8^,^9^ The total cost for Australia was estimated between AU$4.3 and AU$4.9 million in 2007.^9^ In addition, outbreaks of RRV have considerable impacts on tourism, industry and local communities.^10^,^11^

Previous studies have shown spatial heterogeneity of the distribution of RRV disease in one city^12^ and all of Queensland.^13^,^14^ It is important to determine high-risk areas and vulnerable communities so that the government can develop effective intervention strategies and efficiently allocate limited resources. Spatio-temporal models are applicable when data are collected across time and space. Thus, data analysis has to take account of temporal and spatial correlations. Spatio-temporal modeling has received dramatically increased attention in the past few years. The models control the interaction between the spatial component and the temporal component.^15^

Currently, there is a lack of detailed analysis about the contemporary epidemiologic patterns of RRV disease in Queensland. One study analyzed the spatio-temporal trends of RRV outbreaks in Queensland during 1991–2001 at a local government area level and found high occurrence areas in summer and autumn.^16^ The purpose of the current study was to assess spatio-temporal trends of RRV disease during 2001–2011 in Queensland to provide information for developing improved disease control and risk management strategies.

## Materials and Methods

### Study area.

**Figure 1.**
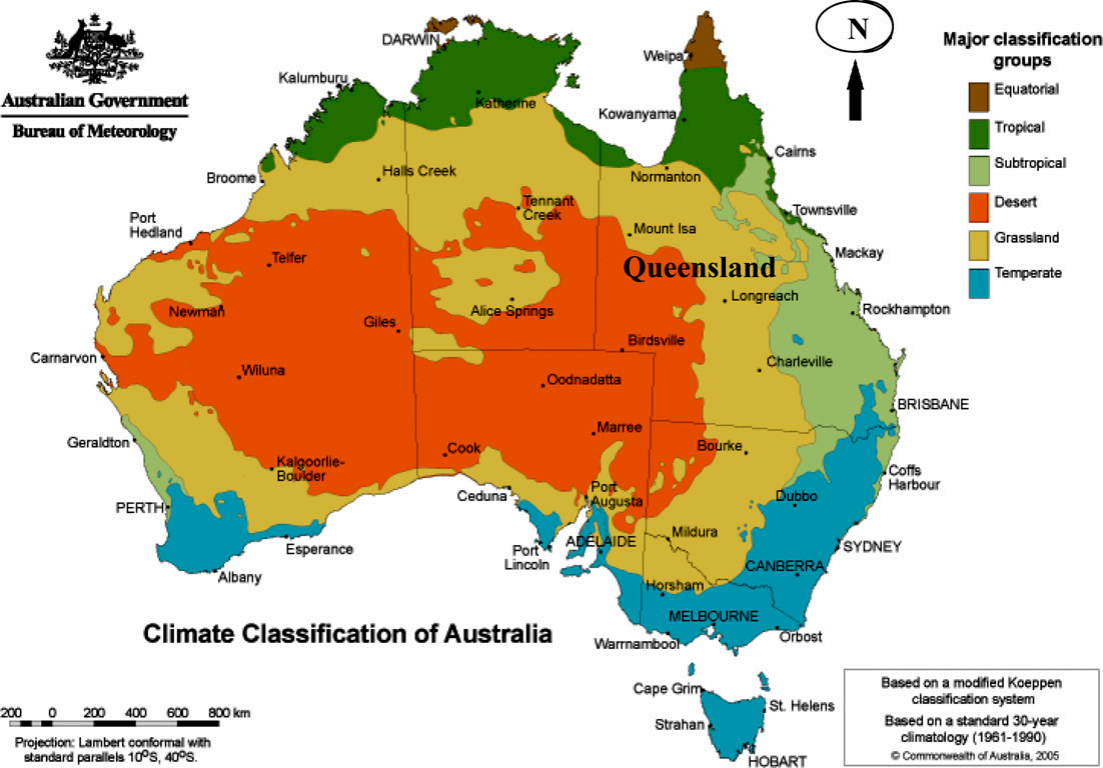
Location and climate of Queensland, Australia.

With an area of 1,727,200 km^2^, Queensland is the second largest state in Australia and is located in the northeastern region of the country. Its climatic environments range widely from tropical rainforest to drought-prone inland deserts (Figure 1). Most of the population lives in the southeastern region of the state and along the eastern coast. Queensland is separated into 478 administrative statistical local areas (SLAs), and these areas range in size from 0.7 km^2^ to 106,188.0 km^2^, and populations range from 7 to 77,544 persons according to the 2006 census.^17^ During the study period (2001–2011), the population increased by approximately 26.2% from 3.63 million to 4.58 million.

### Data collection.

Daily RRV notification data during January 2001–December 2011 were obtained from Queensland Health. Each complete notification included place of residence (SLA code, SLA name), date of onset, date of notification, age group, and sex. A case was confirmed based on a demonstration of IgM against RRV in blood, a ≥ 4-fold change in serum antibody titers between acute-phase and convalescent-phase serum samples, isolation of RRV, or demonstration of arboviral antigen or genome in blood. The annual estimated population for the same period was downloaded from the Australian Bureau of Statistics. Because SLA boundaries change from time to time, we used the 2006 census data to define the SLA boundaries and the 2006 census was conducted in the middle of the study period. All data were converted into the 2006 SLA boundaries for spatial analysis according to area-based correspondence files obtained from the Australian Bureau of Statistics. The monthly counts and incidence rates for each SLA were calculated and stratified by age groups and sex. Direct age- and sex-standardized incidence rates were calculated at a SLA level for spatial analysis. Age- and sex-specific case counts and incidence rates were then summed at a state level.

### Data analysis.

#### Time trend.

We used the annual proportion of monthly RRV cases and Markham's seasonality index to display between-year variation in the data.^18^,^19^ The seasonality index calculates the seasonal concentration of the RRV case load and the peak month in a given year. We stratified the index by age and sex.

### Spatial trend.

#### Spatial visualization.

We mapped the annual age- and sex-standardized incidence rates (SIR) at the SLA level to display the spatial trend during 2001–2011. The SIR for each SLA was calculated by the direct method using the formula

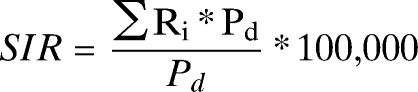


where *R_i_* denotes age- and sex-specific incidence, and *P_d_* denotes age- and sex-specific standard population at each SLA. We selected the estimated age- and sex-specific population in 2006 as the standard population.

### Spatial autocorrelation.

Global Moran's I was used to test the spatial autocorrelation in total and in each year with the following equation:

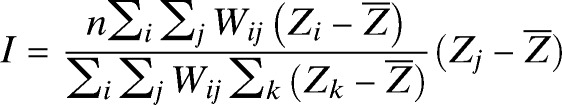


where *Z_i_* and *Z_j_* denote the observed SIRs at SLAs *i* and *j*; 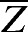 is the average of the Z*_i_* over the n locations, and w*_ij_* is the spatial weight measure of contiguity. The weights matrix was used to define the spatial relationships so that regions close in space are given greater weight when calculating the statistic than those that are distant.^20^,^21^ We used Gabriel graphs to define neighbors.^22^ We assigned a generalized weighting matrix, which was standardized to sum to one. That is, the more neighbors a SLA has, the smaller each individual weight will be compared with SLAs with fewer neighbors.

Three variants of Moran's I statistics were applied as a way to test the sensitivity of our results and check for possible problems with model misspecification. We first tested Moran's I under the assumption of randomness. Next, we used a Monte Carlo simulation to establish the rank of the observed statistic in relation to the simulated values. Finally, we used the Empirical Bayes to provide a form of weighted regression to the mean.

### Spatial variation in risk.

We fitted a conditional autoregressive (CAR) model to predict the total relative risk (RR) and specific RRs in each SLA per year. The smoothed map of predicted RRs was then generated for each year.

The equation of the CAR model, ignoring covariates, is

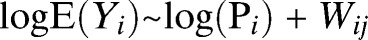


where *Y_i_* denotes the SIR in SLA *i*; *P_i_* denotes the population in SLA *i*, and W*_ij_* is the spatial weights matrix.

### Space-time clustering.

Space-time clustering represents the cases that are close in space and time. The space-time scan statistic is defined by a cylindrical window with a circular (or elliptic) geographic base and with height corresponding to time. At each spatio-temporal location, a cylindrical window increases in size in space and time until it reaches an upper size or population limit. In effect, there are an infinite number of overlapping cylinders of different size and shape, jointly covering the entire study region, where each cylinder reflects a possible cluster. The window with the maximum likelihood is the most likely cluster, that is, the cluster least likely to be caused by chance.^23^,^24^ For the space-time analyses, Software for Spatial and Space-Time Statistics (SaTScan) identifies and orders clusters according to their likelihood ratio test statistic. Usually, the most likely cluster and the secondary clusters are reported.^25^

SaTScan was used to identify the space-time clusters based on a maximum of 50% of the population at risk and the circular special window shape. The results were similar when 10% and 30% of the population were used and an elliptic window shape was used. All analyses were performed in R 3.0.1 (The R Foundation for Statistical Computing, Vienna, Austria, version 3.0.1 http://cran.r-project.org) and SaTScan 9.1.1 (http://www.satscan.org/).

## Results

### General information.

During the study period, 24,425 RRV cases (including 13,025 in females and 11,400 in males) were recorded in Queensland, an average of 185 cases per month (99 in females and 86 in males). The highest number of cases and incidences were among the 40–49 year age group, followed by 30–39, 50–59, and 20–29 year age groups. Only 0.9% and 5% of all cases were reported among children < 10 years of age and the elderly > 70 years of age, respectively (Table 1). The average annual incidence rate was 54 per 100,000 persons (58 for females and 51 for males). The highest and lowest incidence rates were among 40–49- and 0–4-year-old age groups (94 versus 2 per 100,000 persons, respectively).

### Time trends.

**Figure 2.**
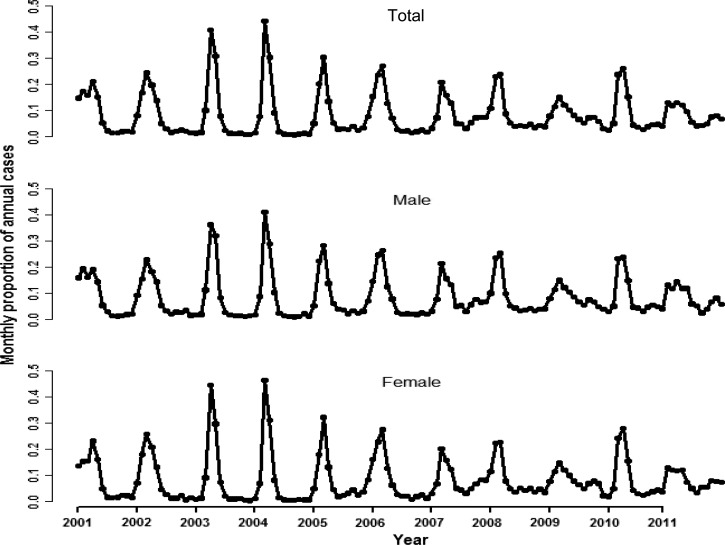
Time trend of monthly case of Ross River virus disease by sex, Queensland, Australia, 2001–2011.

Figure 2 and the complementary figures in the Appendix show that RRV transmission in Queensland is characterized by considerable between- and within-year variations stratified by age and sex. The largest number of RRV cases (3,270) occurred in 2003, with an incidence rate of 85.9 per 100,000 persons. The smallest number was in 2002 with 1,007 cases and an incidence rate of 27.7 per 100,000 persons. The number of cases and corresponding incidence rates were slightly higher for females than males throughout the study period.

The intensity and timing of the seasonal peak also varied from year to year (Supplemental Appendix 1A–1F) stratified by age and sex. The largest monthly proportion of annual cases (0.1–0.5) occurred in the first half of each year, usually during the months of March, April, and May. During 2001–2011, the peak month fluctuated between March and April, with the exception of 2001 for males and 2011 for females, which were characterized by peaks in February and January, respectively. The peak months (March–April) accounted for approximately 21–44% of the total cases (Supplemental Appendix 1A–1F). There was no clear peak month for children and the elderly comparing to the adult groups (Supplemental Appendix 1E).

### Spatial trend.

**Figure 3.**
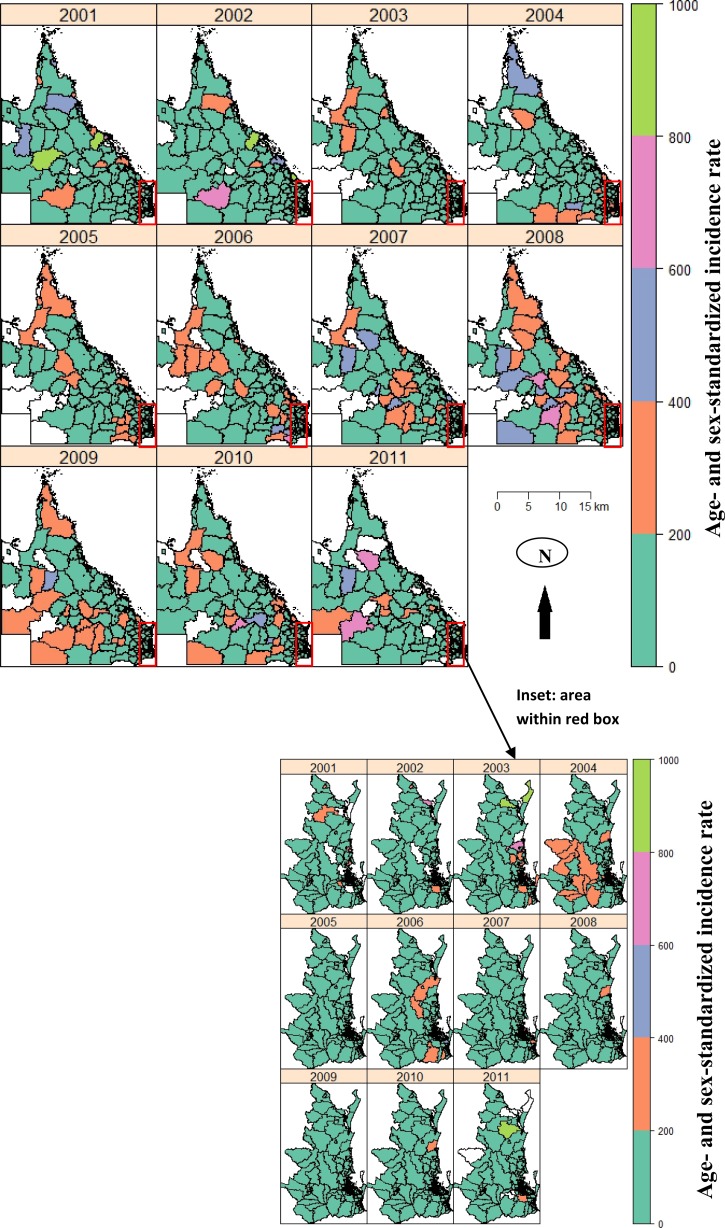
Annual spatial trend of Ross River virus disease in Queensland, Australia, 2001–2011. White color indicates extremly high incidence because of low population.

The spatial distribution of RRV age- and sex-standardized incidence from 2001 to 2011 is shown in Figure 3. Overall, the spatial pattern was clearly different from year to year. Global Moran's I tests showed a spatial variation within each year except in 2001, 2002, and 2011 (Table 2, Supplemental Tables 1 & 2).

**Figure 4.**
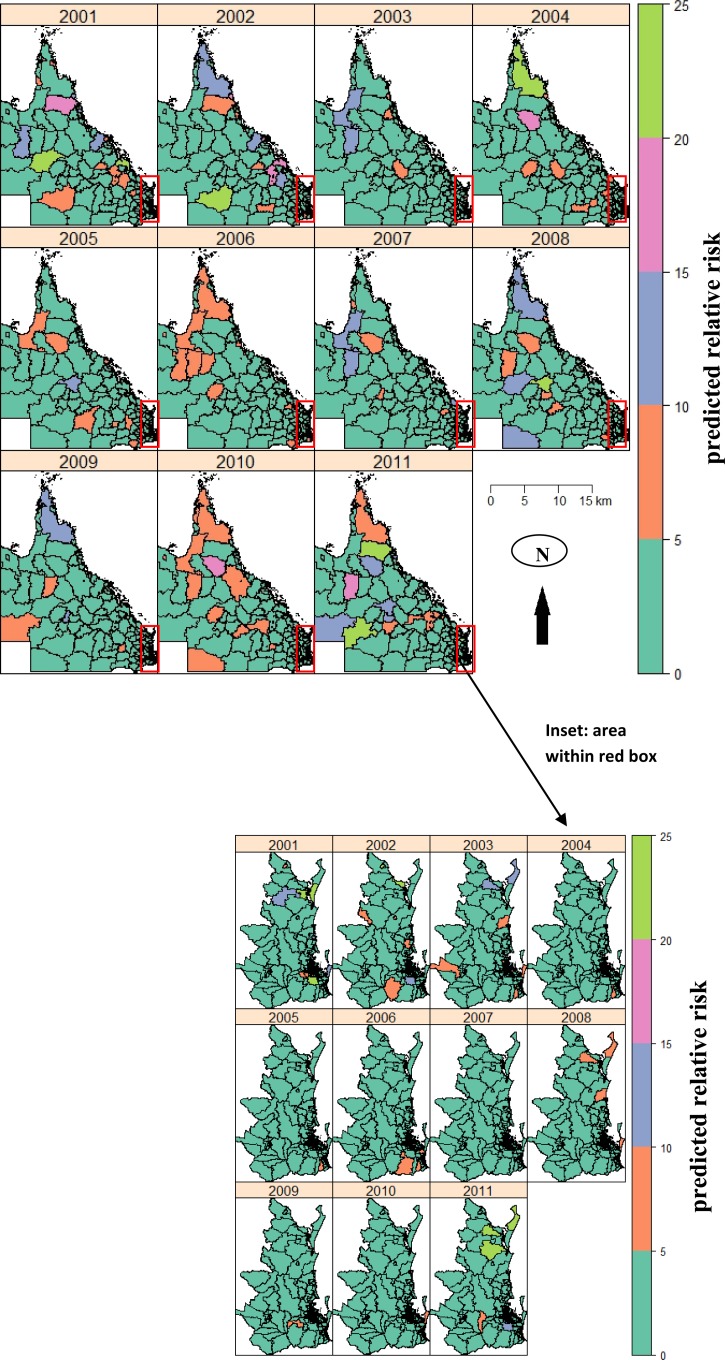
Annual spatial distribution of the predicted relative risk of Ross River virus disease, Queensland, Australia, 2001–2011, by using a conditional autocorrelation model.

A spatially smoothed map displays predicted RRs for each SLA after accounting for autocorrelation using the CAR model for the study period (Figure 4). In the early years of the study period, a small numbers of SLAs were at high infection risks. In the later years, the risk areas expanded to the north, east, and middle of Queensland; most of these SLAs were infected by 2010 and 2011. There was considerable temporal variation in SLA-specific risks over the study period; in particular, most SLAs with a high risk in one year exhibited a low risk in another year. Although the standardized incidences were low for the broader south-eastern area, a few SLAs on the east coast had a high risk but this varied by years (Figure 4).

**Figure 5.**
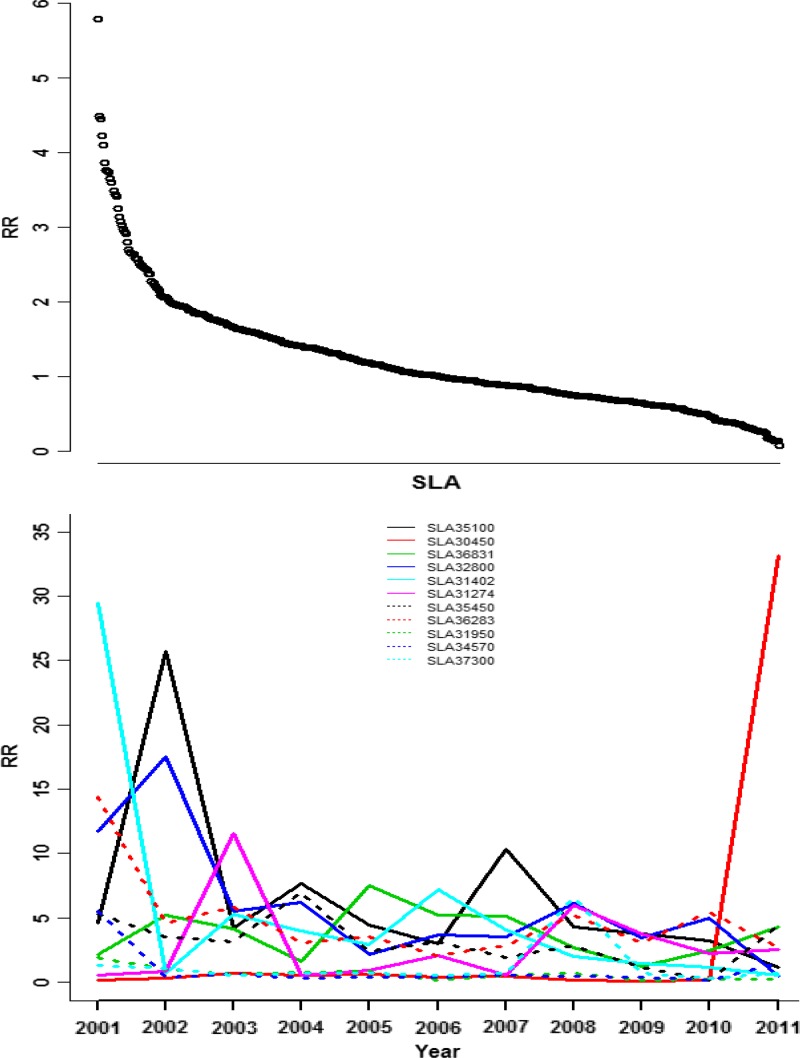
**Top**, Relative risks of Ross River virus (RRV) disease in Queensland, Australia (all statistical local areas [SLAs]). **Bottom**, Time series of the 11 high-risk SLAs using a conditional autocorrelation model.

**Figure 6.**
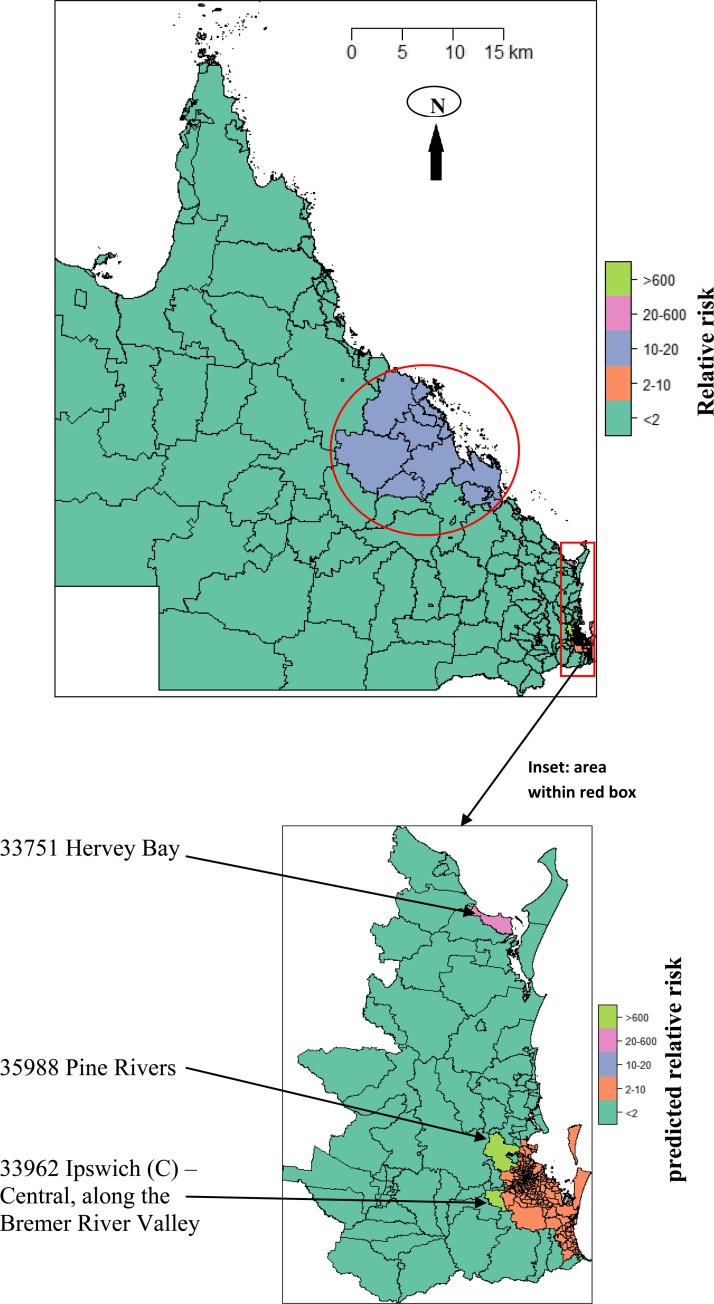
Space-time clusters of Ross River virus risks in Queensland, Australia, 2001–2011.

There were hot spots with higher RRs, although this varied across years (Figure 5

). The RRs in different SLAs indicated different patterns in each year compared with the total RRs. The space-time scan statistics identified two clusters: one around Townsville where the first RRV was isolated, and the other in the mostly densely populated southeastern areas close to the river or the sea, including Pine Rivers and Hervey Bay (Figure 6

).

## Discussion

This study found that there was a clear spatio-temporal trend of RRV distribution and two space-time clusters (i.e., Townsville and the coastal region of southeastern Queensland) were identified during 2001–2011 in Queensland, Australia. The number of RRV annual cases fluctuated during the study period. Peak months fluctuated between March and April. The spatial trend showed a contract-expand pattern during this period.

The distinctive seasonal pattern of the disease is consistent with results of the study by Gatton and others,^16^ and this pattern may be caused by mainly climatic conditions and the life cycle of the vectors and the hosts. Different ecologic characteristics of the more than 40 species of mosquitoes and hosts may be favored as the weather changes. Breeding in coastal intertidal wetlands, *Aedes vigilax* is a significant vector of RRV disease. Other *Aedes* species are vectors breeding in freshwater and are found in many inland areas. *Culex annulirostris* is a major freshwater vector, associated with vegetated semi-permanent and permanent fresh water, and is common in tropical and temperate areas. Marsupials (e.g., kangaroos, wallabies) and other animals (e.g., dogs, cats, horses, and possums) are implicated as intermediate vertebrate hosts for the disease.^26^,^27^ Maximum and minimum temperature, rainfall, relative humidity, and high tides influence the abundance of vectors, hosts, or both.^28^

The results showed a highly variable nature of cases in space and time, and there was clearly high year-to-year variation, even in high-risk SLAs. Seasonal trends and variations between years suggest that climate may be the key determinant of larval and adult mosquito abundance, which subsequently affects RRV transmission. Rainfall is considered the most important climatic factor driving RRV prevalence because of mosquitoes relying on water to complete their life cycle.^11^,^29^,^30^ Gatton and others examined the spatio-temporal patterns of RRV distribution during 1991–2001 in Queensland,^16^ and found 85% of the notifications occurring in the summer and autumn and more than twice as many in the autumn as in the summer. In this study, it was observed that the disease peaked in March and April each year. Previous studies found a two-month lag effect of rainfall on RRV transmission in several cities in Queensland, including Brisbane,^28^,^29^ Townsville,^11^ Cairns,^10^ and the entire state.^28^ The result indicates that summer rain (January and February) may play an important role in disease transmission, which is particularly important for fresh water–breeding mosquitoes in inland areas.^31^ More frequent, lighter rains might replenish existing breeding sites and maintain higher levels of humidity, which assists in dispersal and survival of adult mosquitoes.

Higher temperatures are associated with an increase in the proliferation and reproduction rates of the mosquitoes, an extended transmission season, and many changes in the ecosystems, such as reproduction of the vertebrate host,^31^ migration of vectors, reservoir hosts, or humans.^32^ Relative humidity can directly influence longevity, mating, dispersal, feeding, behavior, and oviposition of the mosquitoes.^11^

The incidence of RRV disease peaked in the 40–49 year age group for both sexes, which is consistent with the national trend. Only 0.9% of the cases occurred in children < 10 years of age, which is similar to that reported nationally because clinically apparent infections are rare in children.^33^,^34^ The cases were more evenly distributed throughout the year, and there were less obvious peak months for children and the elderly. This finding may be caused by the limited number of cases in these age groups and the probability of being less exposed to mosquito bites. We also found that females had slightly higher infection rates than males, which may be explained by sex-differential physiologic factors. Females usually have higher exhaled breath rates and more release of volatile substances from the skin surface than males, which may facilitate mosquito bites.^35^

The pattern of RRV disease incidence varied by region, possibly reflecting the underlying climatic variation, environmental changes, and/or alteration in different vector populations and habitats.^36^ In the southern region, epidemic activity is usually associated with summer and autumn rainfall in temperate inland areas or rain and/or tidal inundation of subtropical coastal marshes during the warmer seasons, when the vectors are most active. In the tropical northern region, distinct seasonal activity associated with the highest spring tides and the wet season is apparent.^27^ Coastal northern Queensland, with little temperature variation and frequent rainfall all year round, contributes to reported RRV infection throughout the year.^26^,^34^,^37^ A study that assessed risk factors across four regions with summer outbreaks in Queensland found that the increased outbreak risk in the southern part of the state was associated with increased spring temperatures and early summer rainfall. In the central coastal region, rainfall was the most important predictor of RRV disease outbreaks. For most of the northern region, rainfall was still the most important factor. However, increased spring temperatures was associated with a decreased risk of outbreak.^38^

The areas around Townsville are still at high risk, which is consistent with results of the study by Gatton and others.^16^ *Aedes vigilax,* whose populations are triggered by high tide events, was the main species in Townsville, where RRV was first isolated from mosquitoes, trapped in 1959 along the Ross River.^31^,^36^,^37^ Adult females lay their eggs on soil, mud, and substrate and at the base of plants around the margins of their breeding sites.^39^ Large populations of adult mosquitoes can emerge in approximately eight days after a series of spring tides, depending on temperature. When the sea level rises, the coastal salt marshes in the region may be inundated more frequently and more extensively. This event might result in extension of breeding grounds for mosquito abundance. The summer population of mosquitoes then may become much larger, and urban dwellers on the eastern seaboard may become exposed more often to the mosquitoes. This hypothesis is crucial to the transmission of RRV infection in the region.^31^

Other factors may also explain the variations in the distribution of RRV disease in Queensland, especially for the two high-risk clustering areas. For example, during warmer months, people spend more time outdoors, further exposing them to RRV transmission. On average, Brisbane residents spend approximately three hours outdoors every day in summer, according to the National Environmental Protection Council report.^40^ Therefore, behavioral changes such as wearing protective clothing and spraying insecticides around the home may protect them against mosquito bites and lower the risk of RRV infection.

Socioeconomic status might be another important contributing factor. People with lower levels of education often have poorer health knowledge about disease transmission and personal protection against mosquito biting.^12^ Higher risk may also be linked to poorer quality of housing among the lower income and socioeconomic groups. Occupation may also be related to transmission. Outdoor workers are likely to be more exposed and have a higher chance of being bitten by mosquitoes. Vegetation density was also associated with RRV disease. Adult mosquitoes may rest in the shade of moderate densities of vegetation, especially where these are close to ovipositon sites.^12^

Population growth and distribution may account for the RRV incidence changes over time and space. During the study period, the population of Queensland increased by 26.2%, much of it in coastal areas, and this was the second fastest growth among all states and territories in Australia.^16^ However, changes in the public health management of RRV disease over time, such as mosquito control and campaigns aimed at increasing public practice of personal protection activities, may reduce human population vulnerability and RRV infection.^41^,^42^ The balance of these two factors will be one of the key determinants of the future transmission of RRV.

This study has several strengths. We used 11 years of data updated until 2011, representing recent trends in RRV transmission. This study displayed time trend and variation within each year among different age and sex groups. We examined incidence at the SLA level, consisting of 478 SLAs in Queensland, which provided spatially detailed information for local governments. Furthermore, we explored the yearly spatial distribution to demonstrate a clear trend during the study period. This feature was different from previous spatial analyses, which averaged several years of data and ignored inter-annual variations. We also used standard incidence to adjust for age and sex heterogeneity to facilitate comparison between SLAs.

Our study also has some limitations. Representativeness is a problem with most surveillance data sets, including RRV. It is quite possible that there are systematic differences between infections that are notified versus those that are not identified and diagnosed. Data are categorized by place of residence. It would be rare (if not impossible) to know where a case acquired the infection. The SLA code changed every year with boundary changes, and there was often a delay in obtaining updated information from the data provider. Also, there may have been some errors when changing the SLA codes to the 2006 boundary using correspondence files.

RRV disease has a strong spatio-temporal pattern in Queensland where the highest number of RRV cases is notified. This study provides detailed epidemiologic information at a SLA level for policymakers, which may assist them to allocate limited resources, in a more effective way, to high-risk areas (e.g., two clusters identified), high-risk seasons (March and April), and most vulnerable groups (persons 40–49 years of age). The seasonal and SLA variations could be useful to local and state authorities to guide preventative measures, such as warning and educating local communities, to develop and implement relevant health promotion campaigns to influence person's behaviors, to avoid activities that expose persons to risk of mosquito bites, and to focus on mosquito control. The information may also provide a basis for building a predictive model that can facilitate future risk assessment and development of an early warning system for preventing and controlling RRV disease outbreaks.

## Supplementary Material

Supplemental Datas.

## Figures and Tables

**Table 1:** Summary for monthly Ross River virus disease cases and yearly incidence stratified by sex and age, Queensland, Australia, 2001–2011

No. monthly cases	Mean (SD)	Minimum	Maximum
Total	185 (222)	15	1333
F	99 (126.2)	4	795
M	86 (97.8)	7	538
0–4	0.4 (0.9)	0	7
5–9	1.2 (1.8)	0	10
10–19	10 (11.6)	0	59
20–29	26 (31.7)	1	162
30–39	43 (57.6)	0	346
40–49	46 (60.6)	1	393
50–59	33 (40.5)	0	251
60–69	17 (17.9)	0	115
70–79	7 (6.0)	0	28
≥ 80	2 (2.0)	0	11
Yearly incidence/100,000)			
Total	54 (18.7)	28	86
F	58 (20.9)	29	93
M	51 (16.8)	26	78
0–4	2 (1.1)	0	4
5–9	5 (2.5)	2	10
10–19	22 (7.2)	11	35
20–29	55 (55.2)	34	80
30–39	87 (32.5)	44	147
40–49	94 (35.3)	45	164
50–59	78 (27.3)	38	128
60–69	56 (22.3)	23	94
70–79	37 (15.0)	15	66
≥ 80	19 (8.6)	6	35

**Table 2:** Spatial autocorrelation of Ross River virus disease cases (Global Moran's I test) in each year and study period (Monte-Carlo simulation of empirical Bayes index), Queensland, Australia, 2001–2011

Year	Observed	Observed rank	*P*
2001	0.009	609	0.39
2002	0.032	819	0.18
2003	0.232	1000	0.001
2004	0.279	1000	0.001
2005	0.375	1000	0.001
2006	0.333	1000	0.001
2007	0.438	1000	0.001
2008	0.335	1000	0.001
2009	0.430	1000	0.001
2010	0.371	1000	0.001
2011	0.002	831	0.17
Total	0.497	1000	0.001
