# Supplementary material for: Epidemiologic Patterns of Ross River Virus Disease in Queensland, Australia, 2001–2011
Source: Am J Trop Med Hyg. 2014 Jul 2;91(1):109–18. doi: 10.4269/ajtmh.13-0455 (PMC4080548; doi:10.4269/ajtmh.13-0455)
Supplement: Supplementary file 1 [file SD3.pdf]

Appendix

1. The time trend of RRV cases among sex and age groups from 2001 to 2011, Queensland, Australia.

A. Monthly proportion by sex.

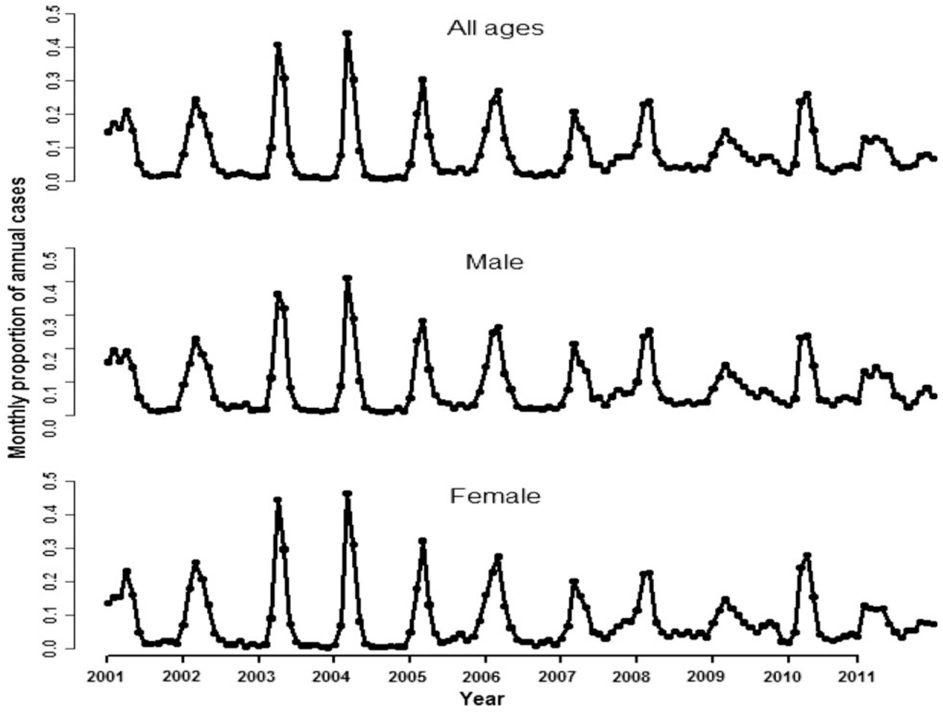

B. Peak month by sex.

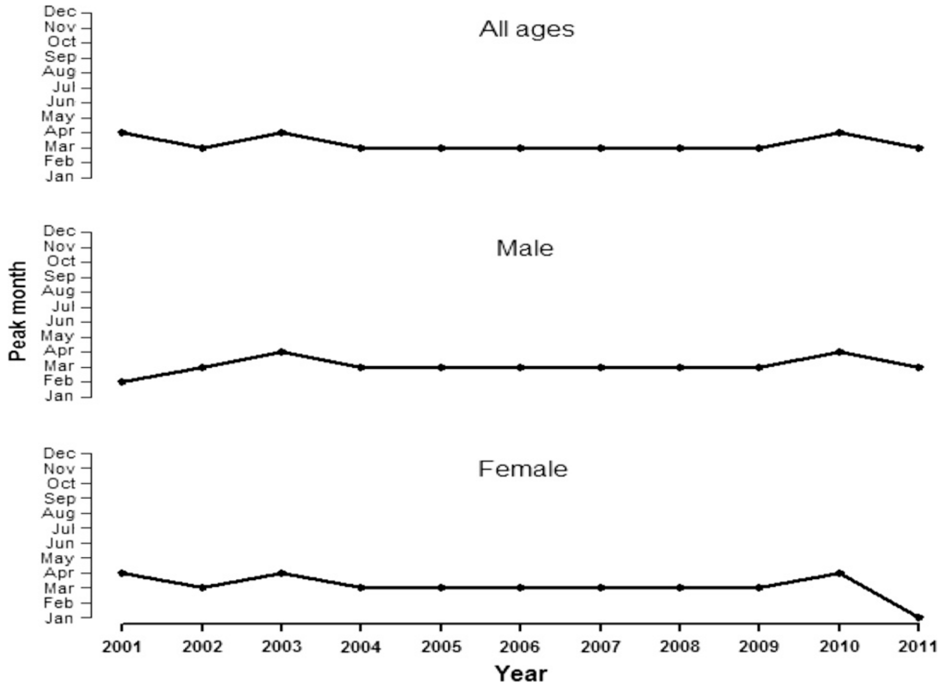

C. % concentration of cases during peak month by sex.

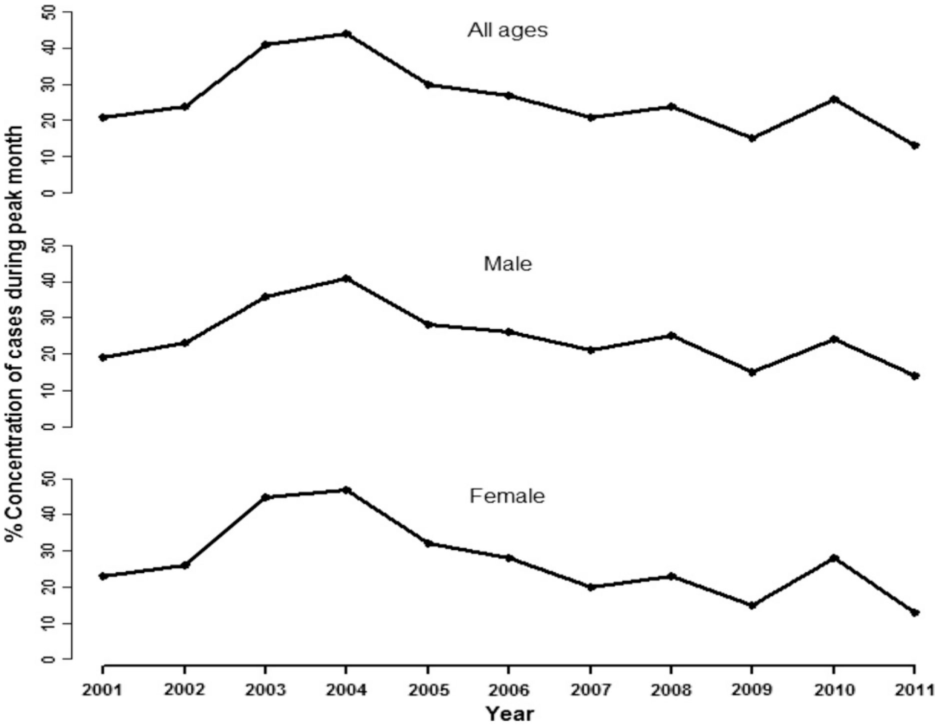

D. Monthly proportion by age.

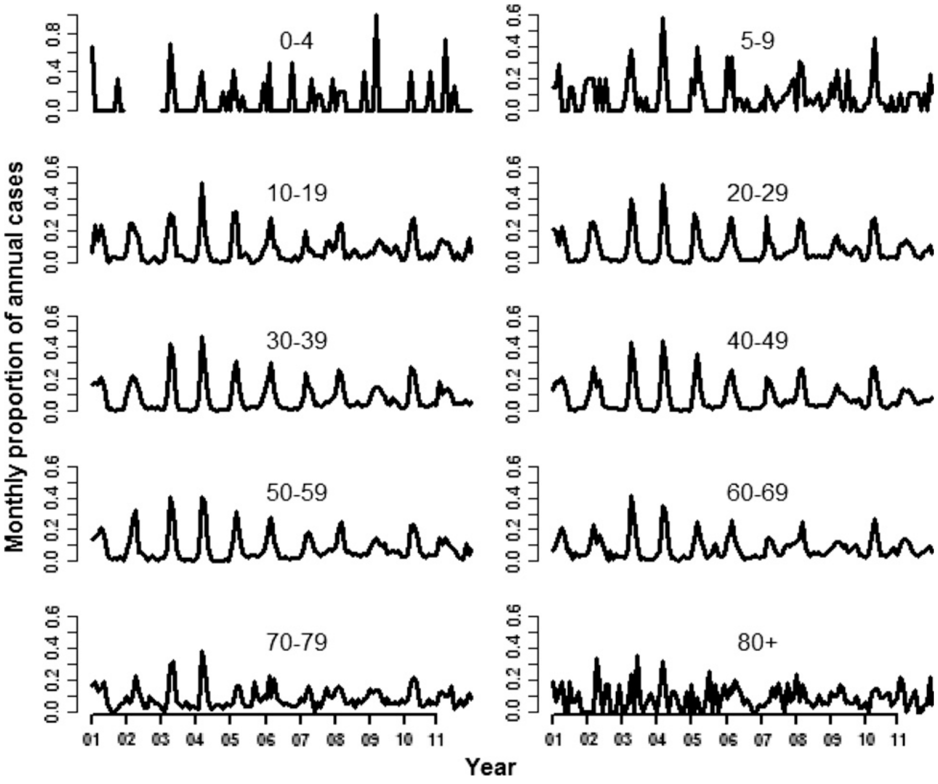

E. Peak month by age.

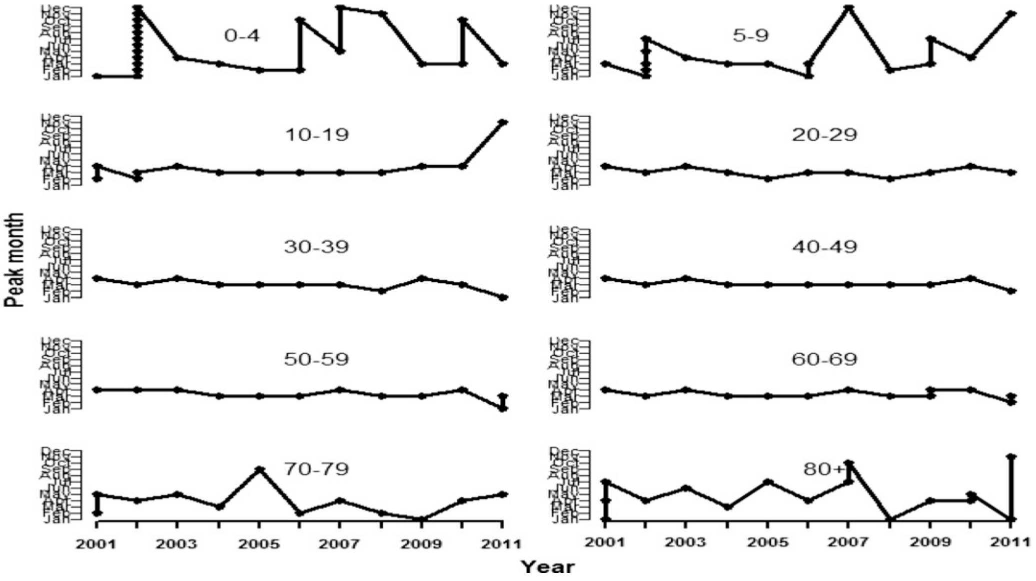

F. % concentration of cases during peak month by age.

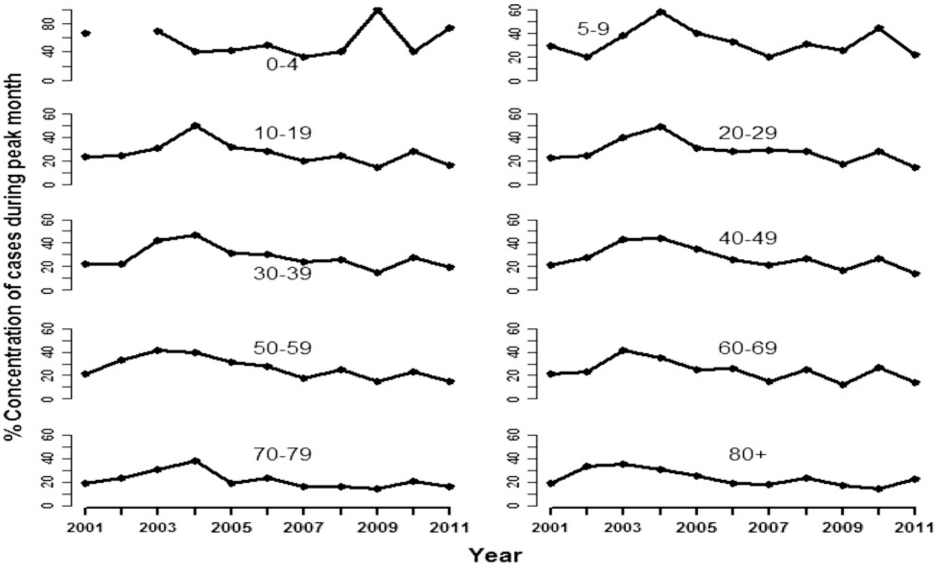

Supplemental Table 1

Spatial autocorrelation of Ross River virus disease cases (Global Moran's I test) in each year and study period (randomness), Queensland, Australia, 2001–2011

Year	Observed	Observed rank	*P*
2001	0.005	750	0.25
2002	0.093	986	0.01
2003	0.185	1000	0.001
2004	0.256	1000	0.001
2005	0.372	1000	0.001
2006	0.295	1000	0.001
2007	0.405	1000	0.001
2008	0.290	1000	0.001
2009	0.434	1000	0.001
2010	0.348	1000	0.001
2011	0.099	992	0.008
Total	0.407	1000	0.001

Supplemental Table 2

Spatial autocorrelation of Ross River virus disease cases (Global Moran' I test) in each year and study period (Monte-Carlo simulation of Moran's I), Queensland, Australia, 2001–2011

Year	Observed	Expected	Variation	SD	*P*
2001	0.015	–0.003	0.0009	0.615	0.27
2002	0.074	–0.003	0.00085	2.625	0.004
2003	0.147	–0.003	0.0009	4.955	3.6×10^{-7}
2004	0.204	–0.003	0.0009	7.241	2.2×10^{-13}
2005	0.295	–0.003	0.0010	9.540	$< 2.2 \times 10^{-16}$
2006	0.234	–0.003	0.0010	7.557	2.1×10^{-14}
2007	0.322	–0.003	0.0010	10.311	$< 2.2 \times 10^{-16}$
2008	0.231	–0.003	0.0010	7.453	4.6×10^{-7}
2009	0.345	–0.003	0.0010	11.037	2.2×10^{-16}
2010	0.276	–0.003	0.0010	8.988	$< 2.2 \times 10^{-16}$
2011	0.079	–0.003	0.0006	3.460	0.0002
Total	0.191	–0.003	0.0010	6.256	1.97×10^{-10}
